# Supplementary material for: Inferring multi-scale neural mechanisms with brain network modelling
Source: eLife. 2018 Jan 8;7:e28927. doi: 10.7554/eLife.28927 (PMC5802851; doi:10.7554/eLife.28927)
Supplement: Supplementary file 1. [file elife-28927-supp1.docx]

| **Fig./**  **p.** | **Test** | **N** | **Definition** | **Descr. Stats.** | **p** | **z** |
| --- | --- | --- | --- | --- | --- | --- |
| 3b | one-tailed  Wilcoxon rank sum  test | 15 | number of subjects  rand.perm. vs hybr.  noise vs hybr.  alpha reg. vs hybr. | mean (--), median, quartiles (boxes), range (whiskers) | p = 0.00036  p = 0.00036  p = 0.0021 | z = -3.38  z = -3.38  z = -2.87 |
| 3d | one-tailed  Wilcoxon rank sum  test | 15 | number of subjects  rand.perm. vs hybr.  RSN1  RSN2  RSN3  RSN4  RSN5  RSN6  RSN7  RSN8  RSN9  noise vs hybr.  RSN1  RSN2  RSN3  RSN4  RSN5  RSN6  RSN7  RSN8  RSN9  alpha reg. vs hybr.  RSN1  RSN2  RSN3  RSN4  RSN5  RSN6  RSN7  RSN8  RSN9 | mean (--), median, quartiles (boxes), range (whiskers) | p = 0.0021  p = 0.0029  p = 0.032  p = 0.0041  p = 0.00098  p = 0.00055  p = 0.44  p = 0.12  p = 0.26  p = 0.00036  p = 0.00036  p = 0.0017  p = 0.0079  p = 0.00036  p = 0.00098  p = 0.4  p = 0.019  p = 0.091  p = 0.05  p = 0.025  p = 0.15  p = 0.037  p = 0.1  p = 0.0092  p = 0.44  p = 0.22  p = 0.88 | z = -2.87  z = -2.75  z = -1.85  z = -2.64  z = -3.1  z = -3.27  z = -0.14  z = -1.16  z = -0.65  z = -3.38  z = -3.38  z = -2.93  z = -2.41  z = -3.38  z = -3.1  z = -0.26  z = -2.07  z = -1.33  z = -1.62  z = -1.96  z = -1.05  z = -1.79  z = -1.28  z = -2.36  z = -0.14  z = -0.77  z = 1.16 |
| 3d | one-tailed  Wilcoxon rank sum  test | 2010 | number of epochs from 15 subjects  hybr. lower quartile vs. hybr. upper quartile  RSN1  RSN2  RSN3  RSN4  RSN5  RSN6  RSN7  RSN8  RSN9  alpha reg. upper quartile vs. hybr. upper quartile  RSN1  RSN2  RSN3  RSN4  RSN5  RSN6  RSN7  RSN8  RSN9  alpha reg. lower quartile vs. hybr. upper quartile  RSN1  RSN2  RSN3  RSN4  RSN5  RSN6  RSN7  RSN8  RSN9 | mean (--), median, quartiles (boxes), range (whiskers) | p < 0.0001  p < 0.0001  p < 0.0001  p < 0.0001  p < 0.0001  p < 0.0001  p < 0.0001  p < 0.0001  p < 0.0001  p < 0.0001  p < 0.0001  p < 0.0001  p < 0.0001  p < 0.0001  p < 0.0001  p = 0.95  p = 0.0022  p = 0.87  p < 0.0001  p < 0.0001  p < 0.0001  p < 0.0001  p < 0.0001  p < 0.0001  p < 0.0001  p < 0.0001  p < 0.0001 | z = -35.85  z = -31.08  z = -37.13  z = -18.47  z = -31.82  z = -37.57  z = -12.70  z = -8.78  z = -14.44  z = -6.43  z = -6.46  z = -12.77  z = -5.44  z = --6.10  z = -11.68  z = 1.67  z = -2.85  z = 1.12  z = -37.46  z = -33.58  z = -38.65  z = -24.05  z = -32.54  z = -38.15  z = -22.64  z = -18.08  z = -8.25 |
| 4a | one-tailed  Wilcoxon rank sum  test | 15 | number of subjects  static FC  rand.perm. vs hybr.  noise vs. hybr.  alpha reg. vs. hybr.  dynamic FC  rand.perm. vs hybr.  noise vs. hybr.  alpha reg. vs. hybr. | mean (--), median, quartiles (boxes), range (whiskers) | p = 0.34  p = 0.019  p = 0.00036  p = 0.13  p = 0.00081  p = 0.00045 | z = -0.43  z = -2.07  z = -3.38  z = -1.11  z = -3.15  z = -3.32 |
| 4b | one-tailed  Wilcoxon rank sum  test | 15 | number of subjects  static FC  rand.perm. vs hybr.  noise vs. hybr.  alpha reg. vs. hybr.  dynamic FC  rand.perm. vs hybr.  noise vs. hybr.  alpha reg. vs. hybr. | mean (--), median, quartiles (boxes), range (whiskers) | p = 0.36  p = 0.00036  p = 0.00036  p = 0.029  p = 0.00036  p = 0.00036 | z = -0.37  z = -3.38  z = -3.38  z = -1.90  z = -3.38  z = -3.38 |
| p. 10 | two-tailed  Wilcoxon rank sum  test | 15 | number of subjects | Cliff’s delta *d* | p = 0.71 | t = 0.54 |
| p. 11 | two-tailed  Wilcoxon rank sum  test | 15 | number of subjects | Cliff’s delta *d* | p = 0.42 | t = -0.2 |
| p. 43 | two-tailed  Wilcoxon rank sum  test | 15 | number of subjects | Cliff’s delta *d* | p = 0.36 | z = -0.37 |
